# Benzoic Acid Used as Food and Feed Additives Can Regulate Gut Functions

**DOI:** 10.1155/2019/5721585

**Published:** 2019-02-26

**Authors:** Xiangbing Mao, Qing Yang, Daiwen Chen, Bing Yu, Jun He

**Affiliations:** ^1^Animal Nutrition Institute, Sichuan Agricultural University, No. 211, Gongpinghuimin Road, Wenjiang District, Chengdu 611130, China; ^2^Key Laboratory of Animal Disease-Resistance Nutrition, Chinese Ministry of Education, Chengdu 611130, China; ^3^Department of Animal Science, Oklahoma State University, Stillwater, OK 74078, USA

## Abstract

As a kind of antibacterial and antifungal preservative, benzoic acid is widely used in foods and feeds. Recently, many studies showed that it could improve the growth and health, which should, at least partially, be derived from the promotion of gut functions, including digestion, absorption, and barrier. Based on the similarity of gut physiology between human and pigs, many relative studies in which piglets and porcine intestinal epithelial cells were used as the models have been done. And the results showed that using appropriate benzoic acid levels might improve gut functions via regulating enzyme activity, redox status, immunity, and microbiota, but excess administration would lead to the damage of gut health through redox status. However, the further mechanisms that some intestinal physiological functions might be regulated are not well understood. The present review will, in detail, summarize the effect of benzoic acid on gut functions.

## 1. Introduction

The gut is a highly proliferative and secretary organ, which plays important functions in the growth and health of the whole body [1]. On the one hand, the intestine is a main organ of nutrient digestion and absorption. On the other hand, it also has the barrier function. This will protect the whole body against pathogens and toxins as the first defense. The gut barrier function is composed of nonspecific barrier mechanisms (including mucosal-epithelial regenerating capacity, intercellular junctions between the epithelial cells, and the mucus gel layer), specific immunological responses, and microbiota [2–8]. Therefore, the gut is important for health of humans and animals, which is associated with nutrient supply and defense from harmful factors.

Because of hypoplasia digestive tract in young children and animals, the production of gastric acid is not enough for the digestion of nutrients (especially protein). This will be not beneficial of growth. And the utilization of acidifiers, including inorganic acidifier, organic acidifier, and complex acidifier, can decrease the pH value in foods and feeds [9]. This can not only increase the nutrient digestibility but also inhibit the harmful microorganisms (such as* E. coli*) in diets and digestive tract [10]. Therefore, the acidifiers have already been considered as a kind of gut health products in humans and animals.

As a kind of organic acidifier, benzoic acid (C_7_H_6_O_2_, BA) is the colorless crystalline solid, which is the simplest aromatic carboxylic acid [11]. It is mainly absorbed and transported in small intestines via the monocarboxylic acid transporter 1 [12]. And the absorption rate for the jejunum is higher than those for the duodenum and ileum, which is consistent with the distribution of monocarboxylic acid transporter 1 [12]. After orally administered [^14^C]BA in man and 20 other species of animal, Bridges et al. (1970) found that, in human, BA would be entirely metabolized to hippuric acid and then be excreted through urine [13].

BA can inhibit pathogenic microorganisms, which makes it a preservative in food and feed industry [14, 15]. It may also increase growth and health and inhibit the diarrhea induced by* E. coli* challenge [16–26]. This can be due to the fact that BA improves the gut functions. Many studies (including the researches in our lab) about the physiological functions of BA mainly involve pigs and porcine cells. However, there is the resemblance between humans and pigs (including gut function and immunity) [27]. Pigs are usually considered as a research model for humans. In addition, intestinal porcine epithelial cell-1 (IPEC-1) is an appropriate model for being directly comparable to the trial animal that is used as an* in vivo* model for humans [28]. Therefore, BA has potential to be used as an additive of improving health in foods.

## 2. Benzoic Acid and Digestion and Absorption in Gut

Dietary BA supplementation can increase the digestibility of nutrients. In recent studies, dietary 0.5% BA supplementation increased the digestibility of total nitrogen, energy, and amino acids in weaned and growing pigs [17, 29–31], and we also found that 0.5% BA administration in diets could increase dry matter, crude protein, ether extract, energy, crush ash, and Ca and P digestibility in weaned piglets (about 1.39-22.38%) and growing pigs (about 2.66-10.38%) [22, 25]. In addition, in lactating sows, the digestibility of organic matter, protein, fat, and fibre is also enhanced by 5.05%, 3.44%, 7.04%, and 34.02% by dietary 2% BA supplementation, respectively [32]. Moreover, Papadomichelakis et al. (2011) showed that the digestibility of organic matter, dry matter, crude protein, energy, and cellulose was increased by dietary 0.5% and 2% BA administration in weaned rabbits [19].

Besides improvement of nutrient digestibility, supplementing BA in diets may decrease the ammonia nitrogen (N) of distal intestinal digesta and faeces. Halas et al. (2010) and Galassi et al. (2011) found that the ammonia-N levels of faeces were reduced 20.17-25.57% by dietary BA supplementation in weaned piglets and finishing pigs [33, 34]. Our recent study in which piglets were utilized as the trial subject also found that BA administration in diets could decrease the ammonia-N concentration (about 49.10-57.32%) in the cecal digesta [23].

**Figure 1:**
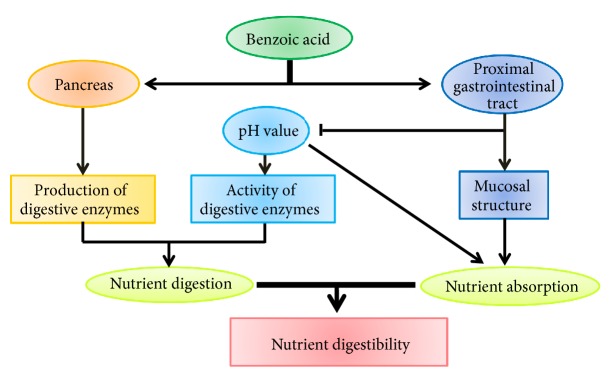
The effect of benzoic acid on nutrient digestibility. Benzoic acid treatment can increase nutrient digestibility in humans and animals via improving nutrient digestion and absorption. This is associated with the improvement of mucosal structure, pH value of digesta, and production and activity of digestive enzymes in intestines.

These results illustrated that BA improves the utilization of nutrients. And it is possible that BA's function should be due to the effect of BA on production and activation of digestive enzymes, and absorption of nutrients in the intestine (Figure 1).

### 2.1. The Effect of BA on Digestive Enzymes

BA administration can promote the production and activation of digestive enzymes. On the one hand, BA increases the digestive enzyme production. Our recent study in which piglets were utilized as the model indicated that the trypsin, lipase, and amylopsin concentrations in the pancreas were increased 74.02%, 67.05%, and 90.44% by BA administration, respectively [35]. On the other hand, BA can activate the digestive enzymes via decreasing the pH value in the proximal gastrointestinal tract. The pH value is important to maintain or increase the activity of enzymes in the gut. In our studies in which pigs were utilized as the trial subject, compared with the control, BA (0.2-0.5%) administration in different-type diets could significantly decrease the pH value of digesta in stomachs (pH 4.14-4.38 vs pH 3.39-3.79) and jejunums (pH 6.15-6.45 vs pH 5.69-6.18) [20, 21, 25, 36]. And the trypsin, lipase, amylase, maltase, sucrose, and lactase activities of digesta were enhanced about 30.02-141.34% by BA treatment in jejunum of piglets, and this effect would be decreased with age [22, 25].

However, Dierick et al. (2004) reported that supplementing 1.0% benzoic acid in the barley-wheat type diet did not significantly affect the pH value in stomach and jejunum of pigs [37]. Therefore, it is possible that the effect of BA on pH value and enzymes' activities in the proximal gastrointestinal tract is associated with dietary type and BA doses.

### 2.2. The Effect of BA on Nutrient Absorption

As the above description, the increasing nutrient digestibility is associated with the improvement of digestive ability. Moreover, the enhancement of nutrient digestibility can also be relative to the absorption capacity. The capacity of absorption mainly depends on the absorbing regions of intestinal mucosa. Thus, the enhancement of surface area in mucosa is beneficial to the substances' transfer from lumen to vascular system [38]. The surface area is mainly associated with gut mucosal structure, such as villi and crypts [38]. Halas et al. (2010) reported that dietary BA supplementation could increase mucosal villus height of ileum in weaned piglets [31]. Our recent studies also showed that BA administration in different-type diets might enhance the villus height and/or decrease the mucosal crypt depth in duodenum, jejunum, and/or ileum of piglets [23, 35, 36]. These results indirectly demonstrate that BA treatment can improve the absorption capacity of the gut.

In addition, it is well known that pH value plays an important role in the mineral absorption. Some studies have shown that BA treatment significantly increases the digestibility of minerals [22, 25, 30], which could be due to the fact that BA administration decreases the pH value of digesta in the gastrointestinal tract.

Therefore, BA has the ability to promote nutrient digestion and absorption in gut, which will lead to the increasing nutrient digestibility in diets. However, some studies also showed that supplementing BA in diets did not promote the nutrient digestibility [16, 34, 39]. Based on the difference among these results, we analyzed and compared the trial design of these researches. And we found that BA improving nutrient digestibility could be influenced by some factors, such as age, dietary type and composition, and environment.

## 3. Benzoic Acid and Gut Barrier Functions

**Figure 2:**
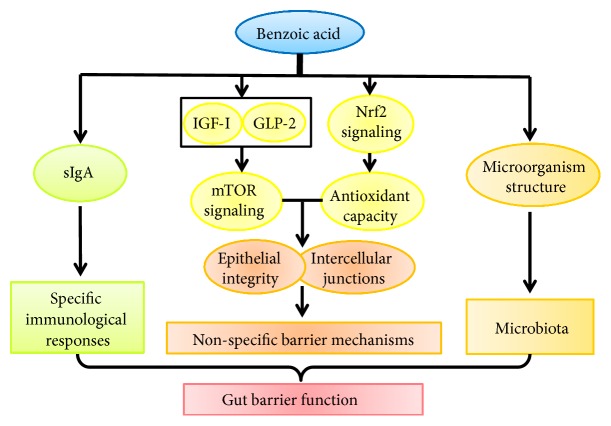
The effect of benzoic acid on gut barrier function. Benzoic acid administration can improve specific immunological responses, nonspecific barrier mechanisms, and microbiota. IGF-I, insulin-like growth factor I. GLP-2, glucagon like peptide 2. Nrf2, nuclear factor-E2-related factor 2. sIgA, secretory immunoglobulin A. mTOR, mammalian target of rapamycin.

BA administration in diets can decrease the serum levels of diamine oxidase and D-lactic acid and alleviate the diarrhea induced by* E. coli* challenge [26, 36], which is possible that BA can increase the barrier functions of intestine. And many studies also further found that BA treatment could indeed improve gut barrier function, including nonspecific barrier mechanisms, specific immunological responses, and microbiota (Figure 2).

### 3.1. The Effect of BA on Nonspecific Barrier Mechanisms in Gut

The mucosal-epithelial integrity is a part of nonspecific barrier mechanisms, which is usually evaluated through morphology analysis [2]. As mentioned above, compared with control, dietary BA supplementation can increase the relative weight (intestinal weight: intestinal length) of small intestine (0.067 vs 0.077), and BA treatment also improves the morphology of the proximal gastrointestinal tract [31], which is similar to our results [23, 35, 36].

The gut can produce many hormones, which will ensure that the gut epithelium has the high ability of regeneration and restitution [40]. The further* in vivo* and* in vitro* experiments in our lab showed that BA treatment could stimulate the expressions of IGF-1 and GLP-2, which regulated some signaling pathways (i.e., mTOR), in small intestine of piglets and IPEC-1 cells [25, 35, 36]. And our* in vivo* and* in vitro* experiments also found that BA treatment improved the redox status in small intestine of piglets and IPEC-1 cells via Nrf2 signaling pathway [35]. These should be the possible reasons that BA improves gut-epithelial integrity.

The intercellular junctions between the epithelial cells also play a critical role for maintaining the gut barrier functions [2]. And the intercellular junctions between the gut epithelial cells are formed mainly via some transmembrane and nonmembrane proteins, including ZO-1 and occludin [41]. The study in which pigs were utilized as the model showed that dietary BA supplementation could stimulate the expressions of ZO-1 and occludin in the jejunal mucosa [36].

These results suggest that BA administration can improve nonspecific barrier mechanisms in the gut. However, it is unknown whether BA treatment can affect the mucus gel layer, as a part of nonspecific barrier mechanisms, in the intestinal mucosa, which needs further researches.

### 3.2. The Effect of BA on Specific Immunological Responses in Gut

There is little study about the effect of BA on gut specific immunological responses. Only a study in which piglets were utilized as the trial subject found that supplementing BA in diets tended to increase the sIgA concentration of duodenum but did not affect that of jejunum and ileum [35]. Except this finding, it is totally unknown whether BA is able to influence cellular immunity and innate immunity (such as host defense peptides) in intestines.

### 3.3. The Effect of BA on Microbiota in Gut

Recently, the critical role of gut microbiota in health, especially gut health, has been gradually recognized [5, 6, 42, 43]. It has been considered as a part of intestinal barrier functions. Torrallardona et al. (2007) and Halas et al. (2010) reported that supplementing BA in diets could increase the diversity of intestinal microbiota in piglets [18, 31]. And 0.5% BA administration can increase population of effective microorganisms (e.g.,* Lactobacillus*,* Bifidobacterium*) and/or decrease population of harmful microorganisms (e.g.,* E. coli*) in the intestine, especially distal intestine [16, 17, 31]. Our recent studies in which piglets were utilized as the model have similar results [21, 23, 24, 36]. However, dietary 0.85% and 1% BA supplementation also decreased the population of* Lactobacillus* while it reduced the population of* E. coli* in the intestinal digesta of pigs [16, 44], and supplementing 0.25-0.75% BA in diets did not affect the gastrointestinal microbiota in broilers [45]. These findings demonstrate that appropriate BA treatment may improve the gut microbiota, and the effect of BA on microbiota is associated with the species of trial subject.

## 4. Excess of Benzoic Acid Administration Impairs the Gut Health

Excessive intake of BA will induce acute or chronic toxicity symptoms, which seriously impairs the health and growth of humans and animals [46–49]. Our recent studies in which piglets were utilized as the model have also shown that excess of dietary BA supplementation leads to the dysfunction and damage of liver, spleen, and lung and may impair the mucosal morphology of duodenum, jejunum, and ileum [50, 51]. In an* in vitro* experiment, excess of BA treatment inhibited the proliferation of IPEC-1 cells, which could be related to the impairment of redox status regulated by Nrf2 pathway [35].

## 5. Conclusion and Future Perspectives

In summary, benzoic acid administration can improve gut functions, including digestion, absorption, and barrier, which is an important reason that benzoic acid can improve the growth and health in the studies that used the animals as models. This is due to the fact that it can regulate enzyme activity, redox status, immunity, and microbiota. Via analysis of results in these studies, we can further ensure that benzoic acid may be used as a kind of gut health products in humans and animals. And it has potential to be used as an additive of improving health in foods, especially to some patients in convalescence. The supplementing dose of BA should be 0.2-0.5% in feeds. However, as gut health products in humans, the using dose of benzoic acid in foods also needs to be further determined. In addition, for further comprehension and utilization, some further researches should be focused on the effect of benzoic acid on gut functions, especially the relative mechanisms.

## References

[1] Turner J. R. (2009). Intestinal mucosal barrier function in health and disease. *Nature Reviews Immunology*.

[2] Mao X., Zeng X., Qiao S., Wu G., Li D. (2011). Specific roles of threonine in intestinal mucosal integrity and barrier function. *Frontiers in Bioscience*.

[3] He F., Wu C., Li P. (2018). Functions and signaling pathways of amino acids in intestinal inflammation. *BioMed Research International*.

[4] Wang S., Chen Y.-G. (2018). BMP signaling in homeostasis, transformation and inflammatory response of intestinal epithelium. *SCIENCE CHINA Life Sciences*.

[5] Zhu D., Ma Y., Ding S., Jiang H., Fang J. (2018). Effects of melatonin on intestinal microbiota and oxidative stress in colitis mice. *BioMed Research International*.

[6] Chen F., Jiang J., Tian D. (2017). Targeting obesity for the prevention of chronic cardiovascular disease through gut microbiota-herb interactions: An opportunity for traditional herbs. *Current Pharmaceutical Design*.

[7] Eri R., Vemuri R., Gundamaraju R. (2017). Editorial: novel interventional targets for gastrointestinal and metabolic disorders. *Current Pharmaceutical Design*.

[8] Weichselbaum L., Klein O. D. (2018). The intestinal epithelial response to damage. *Science China Life Sciences*.

[9] Diao H. (2013). *Effects of Benzoic Acid and Thymol on Growth Performance and Gut Health in Piglets*.

[10] Qin S., Zhang H., Tang X., Wang Y. (2007). The physiology function of acidifier and how to select acid from different acids. *Chinese Journal of Animal Nutrition*.

[11] Sim G. A., Robertson J. M., Goodwin T. H. (1955). The crystal and molecular structure of benzoic acid. *Acta Crystallographica*.

[12] Cong D., Fong A. K. Y., Lee R., Pang K. S. (2001). Absorption of benzoic acid in segmental regions of the vascularly perfused rat small intestine preparation. *Drug Metabolism and Disposition*.

[13] Bridges J. W., French M. R., Smith R. L., Williams R. T. (1970). The fate of benzoic acid in various species.. *Biochemical Journal*.

[14] del Olmo A., Calzada J., Nuñez M. (2015). Benzoic acid and its derivatives as naturally occurring compounds in foods and as additives: Uses, exposure, and controversy. *Critical Reviews in Food Science and Nutrition*.

[15] Knarreborg A., Miquel N., Granli T., Jensen B. B. (2002). Establishment and application of an in vitro methodology to study the effects of organic acids on coliform and lactic acid bacteria in the proximal part of the gastrointestinal tract of piglets. *Animal Feed Science and Technology*.

[16] Kluge H., Broz J., Eder K. (2006). Effect of benzoic acid on growth performance, nutrient digestibility, nitrogen balance, gastrointestinal microflora and parameters of microbial metabolism in piglets. *Journal of Animal Physiology and Animal Nutrition*.

[17] Guggenbuhl P., Séon A., Quintana A. P., Nunes C. S. (2007). Effects of dietary supplementation with benzoic acid (VevoVitall®) on the zootechnical performance, the gastrointestinal microflora and the ileal digestibility of the young pig. *Livestock Science*.

[18] Torrallardona D., Badiola I., Broz J. (2007). Effects of benzoic acid on performance and ecology of gastrointestinal microbiota in weanling piglets. *Livestock Science*.

[19] Papadomichelakis G., Mountzouris K. C., Zoidis E., Fegeros K. (2011). Influence of dietary benzoic acid addition on nutrient digestibility and selected biochemical parameters in fattening rabbits. *Animal Feed Science and Technology*.

[20] Chen J., Chen D., Yu B. (2015). Effects of benzoic acid on growth performance, organ indexes and gastrointestinal content pH of weaned piglets. *Chinese Journal of Animal Nutrition*.

[21] Gao Z., Yu B., Zheng P. (2014). Effects of Benzoic acid on intestinal microflora and metabolites of piglets. *Chinese Journal of Animal Nutrition*.

[22] Diao H., Zheng P., Yu B. (2013). Effects of benzoic acid on growth performance, serum biochemical parameters, nutrient digestibility and digestive enzyme activities of jejunal digesta in weaner piglets. *Chinese Journal of Animal Nutrition*.

[23] Diao H., Zheng P., Yu B. (2014). Effects of dietary supplementation with benzoic acid on intestinal morphological structure and microflora in weaned piglets. *Livestock Science*.

[24] Diao H., Zheng P., Yu B. (2015). Effects of benzoic acid and thymol on growth performance and gut characteristics of weaned piglets. *Asian-Australasian Journal of Animal Sciences*.

[25] Diao H., Gao Z., Yu B. (2016). Effects of benzoic acid (VevoVitall®) on the performance and jejunal digestive physiology in young pigs. *Journal of Animal Science and Biotechnology*.

[26] Halas D., Hansen C. F., Hampson D. J., Mullan B. P., Wilson R. H., Pluske J. R. (2009). Effect of dietary supplementation with inulin and/or benzoic acid on the incidence and severity of post-weaning diarrhoea in weaner pigs after experimental challenge with enterotoxigenic Escherichia coli. *Archives of Animal Nutrition*.

[27] Mao X., Gu C., Ren M. (2018). l-Isoleucine Administration Alleviates Rotavirus Infection and Immune Response in the Weaned Piglet Model. *Frontiers in Immunology*.

[28] Verhoeckx K., Cotter P., López-Expósito I. (2015). *The Impact of Food Bioactives on Health*.

[29] Bühler K., Bucher B., Wenk C., Broz J. (2009). Influence of benzoic acid in high fibre diets on nutrient digestibility and VFA production in growing/finishing pigs. *Archives of Animal Nutrition*.

[30] Sauer W., Cervantes M., Yanez J. (2009). Effect of dietary inclusion of benzoic acid on mineral balance in growing pigs. *Livestock Science*.

[31] Halas D., Hansen C. F., Hampson D. J. (2011). Dietary supplementation with benzoic acid improves apparent ileal digestibility of total nitrogen and increases villous height and caecal microbial diversity in weaner pigs. *Animal Feed Science and Technology*.

[32] Kluge H., Broz J., Eder K. (2010). Effects of dietary benzoic acid on urinary pH and nutrient digestibility in lactating sows. *Livestock Science*.

[33] Halas D., Hansen C. F., Hampson D. J. (2010). Effects of benzoic acid and inulin on ammonia-nitrogen excretion, plasma urea levels, and the pH in faeces and urine of weaner pigs. *Livestock Science*.

[34] Galassi G., Malagutti L., Colombini S., Rapetti L., Matteo Crovetto G. (2011). Effects of benzoic acid on nitrogen, phosphorus and energy balance and on ammonia emission from slurries in the heavy pig. * Italian Journal of Animal Science*.

[35] Gao Z. (2013). *Regulatory Effects of Benzoic Acid on Digestive Physiology and Nutritional Metabolism of Young Pigs*.

[36] Chen J. (2015). *Effects of Benzoic Acid on Growth Performance and Intestinal Function and the Optimum Dietary Dose in Weaning Piglets*.

[37] Dierick N., Michiels J., Van Nevel C. (2004). Effect of medium chain fatty acids and benzoic acid, as alternatives for antibiotics, on growth and some gut parameters in piglets. *Communications in Agricultural and Applied Biological Sciences*.

[38] DeSesso J. M., Jacobson C. F. (2001). Anatomical and physiological parameters affecting gastrointestinal absorption in humans and rats. *Food and Chemical Toxicology*.

[39] Bühler K., Bucher B., Wenk C. (2010). Apparent nutrient and mineral digestibility in growing-finishing pigs fed phosphorus reduced diets supplemented with benzoic acid and phytase. *Livestock Science*.

[40] Dignass A. U. (2001). Mechanisms and modulation of intestinal epithelial repair. *Inflammatory Bowel Diseases*.

[41] Laukoetter M. G., Bruewer M., Nusrat A. (2006). Regulation of the intestinal epithelial barrier by the apical junctional complex. *Current Opinion in Gastroenterology*.

[42] Diao H., Yan H. L., Xiao Y. (2016). Erratum to: Intestinal microbiota could transfer host Gut characteristics from pigs to mice. *BMC Microbiology*.

[43] Yan H., Diao H., Xiao Y. (2016). Gut microbiota can transfer fiber characteristics and lipid metabolic profiles of skeletal muscle from pigs to germ-free mice. *Scientific Reports*.

[44] Øverland M., Kjos N. P., Borg M., Sørum H. (2007). Organic acids in diets for entire male pigs. *Livestock Science*.

[45] Józefiak D., Kaczmarek S., Bochenek M., Rutkowski A. (2007). A note on effect of benzoic acid supplementation on the performance and microbiota population of broiler chickens. *Journal of Animal and Feed Sciences*.

[46] Amaechi N., Anueyiagu C. (2012). The effect of dietary benzoic acid supplementation on growth performance and intestinal wall morphology of broilers. *Online Journal of Animal and Feed Research*.

[47] Bedford P. G., Clarke E. G. (1972). Experimental benzoic acid poisoning in the cat.. *Veterinary Record*.

[48] qi P., Hong H., Liang X., Liu D. (2009). Assessment of benzoic acid levels in milk in China. *Food Control*.

[49] Andersen F. A. (2001). Final report on the safety assessment of Benzyl Alcohol, Benzoic Acid, and Sodium Benzoate. *International Journal of Toxicology*.

[50] Shu Y., Yu B., He J. (2016). Excess of dietary benzoic acid supplementation leads to growth retardation, hematological abnormality and organ injury of piglets. *Livestock Science*.

[51] Shu Y. (2016). *Safety Evaluation of Benzoic Acid in Piglets*.

